# Skene’s Gland Malignancy from Imaging Diagnosis to Management: A Narrative Review

**DOI:** 10.3390/diagnostics16193213

**Published:** 2026-10-03

**Authors:** Roxana-Denisa Capraș, Teodora Telecan, Lucas Tellier, Miana Gabriela Pop, Adriana Ioana Gaia-Oltean

**Affiliations:** 1 Department of Anatomy and Embryology, “Iuliu Hațieganu” University of Medicine and Pharmacy, 400012 Cluj-Napoca, Romania; capras.roxana@umfcluj.ro (R.-D.C.); lucas.tellier12@gmail.com (L.T.); 2 Department of Public Health and Management, Faculty of Medicine, “Iuliu Hațieganu” University of Medicine and Pharmacy, Victor Babeș Str., No. 8, 400012 Cluj-Napoca, Romania; dr.miana.hosu@gmail.com; 3 Department of Obstetrics and Gynecology, “Iuliu Hațieganu” University of Medicine and Pharmacy, 400347 Cluj-Napoca, Romania; oltean_adriana_ioana@yahoo.com

**Keywords:** Skene’s gland malignancy, paraurethral tumor, pelvic MRI, female urethral adenocarcinoma, differential diagnosis, clinical presentation

## Abstract

**Background/Objectives:** Skene’s gland malignancies are extremely rare tumors of the female paraurethral region, known almost exclusively from case reports and small case series. Their diagnosis is often delayed because symptoms are nonspecific and may mimic benign periurethral disorders, urethral diverticula, primary urethral carcinoma or anterior vaginal wall tumors. This narrative review focuses on the role of imaging, particularly pelvic magnetic resonance imaging (MRI), in the diagnosis, differential diagnosis, staging and treatment planning of Skene’s gland malignancies, distinguishing evidence from confirmed Skene’s gland cases from evidence extrapolated from female urethral carcinoma. **Methods:** PubMed/MEDLINE, Scopus and Web of Science were searched on 27 September 2026 (publication years 2015–2026) and supplemented by reference-list screening; an explicit case definition was applied. **Results:** Of 191 records (93 after de-duplication), 17 reports of Skene’s gland malignancy were included (15 case reports or series and 2 systematic reviews); fewer than thirty cases have been published overall. Patients were mostly postmenopausal (median age about 70 years, range 45–91), presenting with an asymptomatic periurethral nodule, hematuria or vaginal bleeding, or lower urinary tract symptoms; no uniform staging system has been applied. Most tumors were prostatic-type adenocarcinomas treated by local excision alone; radiotherapy, chemotherapy or androgen deprivation were used mainly for nodal, metastatic or recurrent disease. After examination, ultrasonography can detect and characterize a paraurethral mass, whereas MRI best defines tumor epicenter, urethral, periurethral and anterior vaginal wall involvement and regional spread, and should precede cystourethroscopy and biopsy; CT and PET/CT are complementary for systemic staging. No single immunohistochemical marker establishes Skene’s gland origin. **Conclusions:** MRI is central to localizing and staging suspected Skene’s gland malignancy and thus to guiding biopsy and the choice between local excision and radical surgery, but most imaging recommendations are extrapolated from female urethral carcinoma and require validation in pooled multicenter data.

## 1. Introduction

The Skene’s glands, also known as paraurethral glands, are homologous to the male prostate and are located adjacent to the distal female urethra. Although benign inflammatory and cystic disorders of the Skene’s glands are relatively uncommon, malignant transformation is exceedingly rare. Skene’s gland malignancies are generally classified among female urethral adenocarcinomas, representing a minute fraction of genitourinary cancers in women.

The rarity of these tumors has resulted in a limited understanding of their natural history, optimal imaging work-up, prognostic factors, and therapeutic management. Most available data derive from isolated case reports and small retrospective series. Consequently, diagnosis is frequently delayed because symptoms such as dysuria, dyspareunia, hematuria, urinary obstruction, or recurrent urinary tract infections are nonspecific and may mimic benign periurethral lesions.

Reliable population-based epidemiological data specific to Skene’s gland malignancy are lacking, given the extreme rarity of this tumor. Population-based cancer registry data indicate that primary urethral carcinoma itself is an uncommon malignancy in women, with an estimated age-adjusted incidence below two cases per million population, increasing markedly among women older than 75 years [1]. Urethral carcinoma accounts for approximately 0.02% of all female cancers and 0.003% of female urogenital malignancies, adenocarcinoma represents only 8–10% of primary female urethral carcinomas, and female urethral adenocarcinoma as a whole is associated with a poor prognosis and low survival [2,3]. Within this already rare group of tumors, neoplasms arising from the Skene’s glands are considered exceptionally uncommon: depending on the diagnostic criteria applied, between 15 and 22 cases had been collected by successive reviews up to 2023, and fewer than thirty cases have been published worldwide to date, with a median patient age around the seventh decade of life (Section 4.1) [2,4,5]. Current knowledge of this entity therefore continues to rely almost entirely on isolated case reports and small case series rather than on population-level data.

Advances in pelvic imaging and immunohistochemical characterization have improved the recognition of periurethral neoplasms. MRI, diffusion-weighted imaging and contrast-enhanced sequences are increasingly used for the local staging and surgical planning of periurethral tumors, although their use in Skene’s gland malignancy is documented in only a few reports [2,6,7]. Concurrently, immunohistochemical studies have reinforced the concept that Skene’s gland derivatives and some of their tumors share an immunophenotype with prostatic tissue [8,9].

This narrative review aims to summarize the current evidence on Skene’s gland malignancies with a particular focus on the role of imaging in their diagnosis, differential diagnosis, local staging and treatment planning, explicitly distinguishing evidence obtained from confirmed Skene’s gland cases from evidence extrapolated from female urethral carcinoma.

## 2. Materials and Methods

### 2.1. Search Strategy and Study Selection

This manuscript was prepared as a narrative review based on a structured and reproducible literature search, addressing the diagnosis, imaging assessment, differential diagnosis and therapeutic approach of Skene’s gland malignancies. PubMed/MEDLINE, Scopus and the Web of Science Core Collection were searched; all searches were last run on 27 September 2026 and were limited to publication years 2015–2026.

In each database, two search strings were used: (i) Skene’s gland AND malignancy and (ii) Skene’s gland AND imaging, adapted to the syntax of each database (PubMed: (skene’ gland) AND (malignancy) and (skene’s glands) AND (imaging); Scopus: TITLE-ABS-KEY(“skene* gland*” AND malignan*) and TITLE-ABS-KEY(“skene* gland*” AND imaging); Web of Science: TS = (“skene* gland*” AND malignan*) and TS = (“skene* gland*” AND imaging)). To identify case reports published before 2015 or not retrieved by these strings, and to collect supporting literature, the reference lists of the included articles and of previous reviews [2,4,5] were screened, and additional targeted searches were performed using the terms “female paraurethral adenocarcinoma”, “female urethral adenocarcinoma”, “female urethral carcinoma”, “periurethral tumor”, “paraurethral mass”, “female urethral MRI”, “periurethral tumor MRI”, “ultrasonography”, “computed tomography”, “PET/CT”, “histopathology” and “immunohistochemistry”.

Records were de-duplicated and screened by title and abstract, followed by full-text assessment. For the case-level synthesis (Table 1), reports were eligible if they described one or more patients with a primary malignancy attributed to the Skene’s (paraurethral) glands, or systematically reviewed such cases. Female urethral adenocarcinomas for which a Skene’s gland origin was neither claimed nor supported were excluded from the case count and retained as comparators. Articles published in English, or in other languages with an informative English abstract, were considered. Duplicate reports of the same patient were sought by comparing age, presentation, institution and treatment across individual reports and previous reviews [2,4,5]; no duplicate patients were identified among the individually tabulated cases. Supporting literature on anatomy, imaging, pathology, differential diagnosis and treatment of female urethral and periurethral lesions was selected narratively from the same searches and from reference lists; studies dealing only with male urethral or prostatic malignancies were considered only when relevant to the homology between the Skene’s glands and the prostate or to the interpretation of prostate-associated immunohistochemical markers.

**Figure 1:**
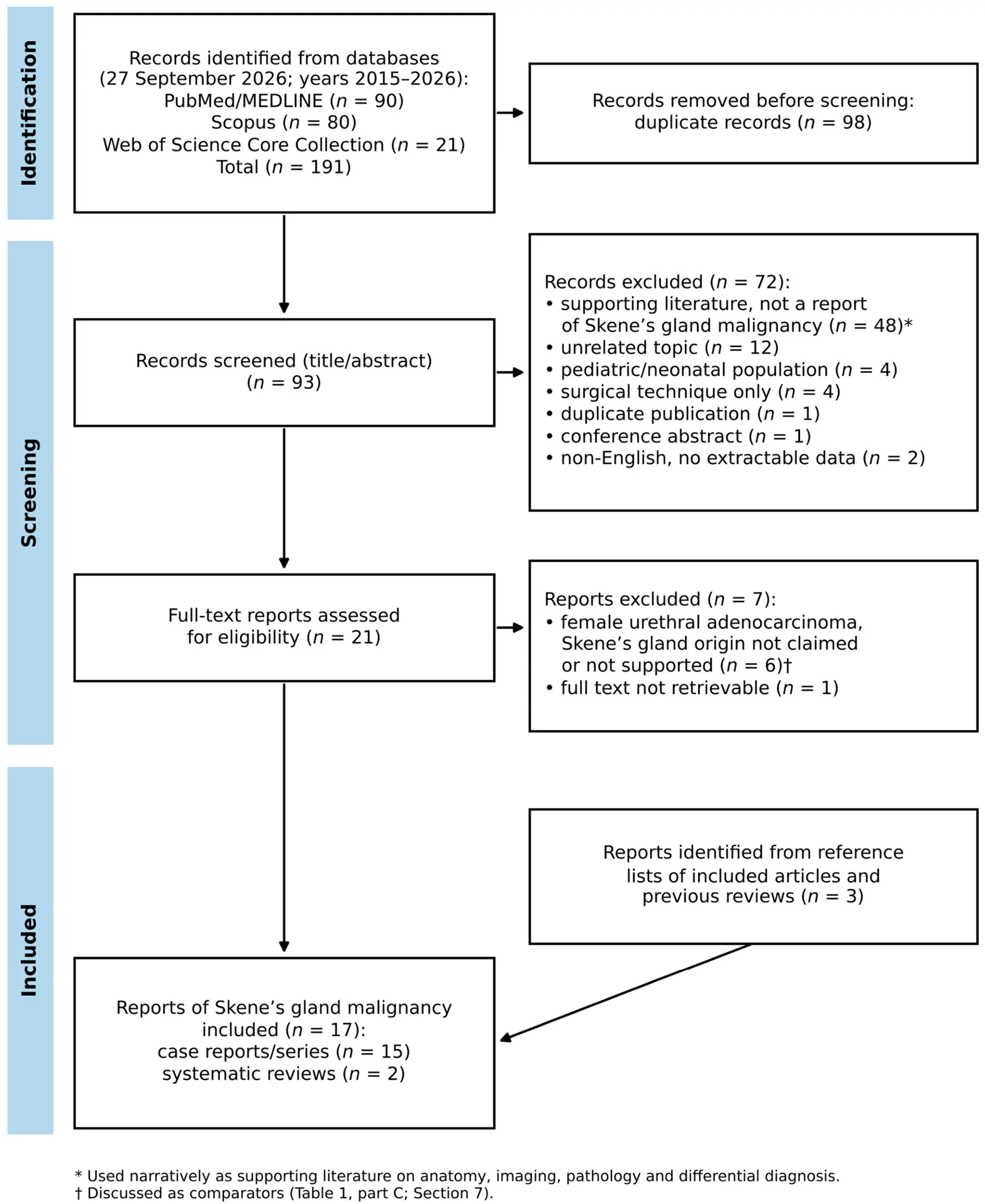
Flow diagram of the literature search and selection of reports of Skene’s gland malignancy (adapted from the PRISMA 2020 flow diagram). Records excluded as “supporting literature” were used narratively for the sections on anatomy, imaging, pathology and differential diagnosis.

The database searches yielded 191 records (PubMed 90, Scopus 80, Web of Science 21). After removal of 98 duplicates, 93 unique records were screened, of which 72 were excluded at the title/abstract stage: 48 addressed benign periurethral disease or the imaging, pathology or management of female urethral lesions without reporting a Skene’s gland malignancy and were used as supporting literature, and 24 were unrelated to the topic, concerned pediatric patients or surgical technique only, or were duplicate publications, conference abstracts or non-English reports without extractable data. Of 21 full-text reports assessed, six female urethral adenocarcinomas without a claimed or demonstrable Skene’s gland origin and one report whose full text could not be retrieved were excluded, leaving 14 reports; three further case reports were identified from reference lists, for a total of 17 included reports (Figure 1).

### 2.2. Case Definition

In the absence of accepted diagnostic criteria, a tumor was classified as a Skene’s gland primary when all of the following were met: (i) a lesion centered in the periurethral/paraurethral tissues (around the distal or mid urethra, the external urethral meatus or the bladder neck and proximal urethra), as documented clinically, radiologically, endoscopically or pathologically; (ii) no evidence of another primary tumor that could account for the lesion by direct extension or metastasis (in particular colorectal, endometrial or cervical, vaginal, bladder or renal primaries); and (iii) either predominant prostatic-type differentiation (morphology resembling prostatic acinar or ductal adenocarcinoma with expression of prostate-specific antigen (PSA), prostate-specific acid phosphatase (PSAP), NKX3.1 or P501S/prostein) or morphological or immunohistochemical evidence of origin from native paraurethral glands, such as an in situ or prostatic intraepithelial neoplasia (PIN)-like precursor, a transition from benign Skene’s glands, or preserved basal cells demonstrated with a PIN4/p63 cocktail [8,10,11,12]. Tumors attributed to the Skene’s glands by the original authors that fulfilled criteria (i) and (ii) but showed a non-prostatic (intestinal, mucinous, adenosquamous or mixed) phenotype, or for which immunophenotypic data were insufficient, without demonstrated origin from native glands, were classified as probable Skene’s gland primaries and are tabulated separately (Table 1, part B).

### 2.3. Evidence Classification and Synthesis

Because direct evidence is scarce, statements and recommendations were classified according to their source as: (D) direct evidence from reported Skene’s gland malignancies; (E) evidence extrapolated from female urethral carcinoma, female urethral adenocarcinoma or benign periurethral lesions; or (X) expert inference based on anatomy and tumor extent. This classification is indicated in the text where relevant and summarized in Section 8. Because the available evidence is limited, heterogeneous and largely based on isolated cases or small series, no quantitative analysis or formal risk-of-bias assessment was performed, and the data were synthesized descriptively, with emphasis on clinical, imaging-based and pathological diagnostic evaluation and multidisciplinary decision-making.

During the preparation of this manuscript, the authors used Claude Opus 5.5 (Anthropic, San Francisco, CA, USA) to assist with executing and documenting the literature searches, preliminary screening, literature synthesis, drafting and reference formatting. All search results, screening decisions, source material, and clinical and imaging data were verified by the authors against the original publications, and the authors take full responsibility for the accuracy and content of this manuscript.

## 3. Anatomy and Embryological Background

The Skene’s glands are paired paraurethral glands situated near the distal urethra and opening close to the external urethral meatus. These secrete mucus through ducts opening near the external urethral meatus [13].

They originate embryologically from the urogenital sinus and are considered homologous to the male prostate gland. This embryological similarity explains the immunophenotypic overlap observed in Skene’s gland adenocarcinomas.

The periurethral location of these glands has significant implications for both diagnosis and treatment. Tumors may infiltrate the urethra, anterior vaginal wall, bladder neck, or periurethral soft tissues, complicating surgical excision and local staging.

**Figure 2:**
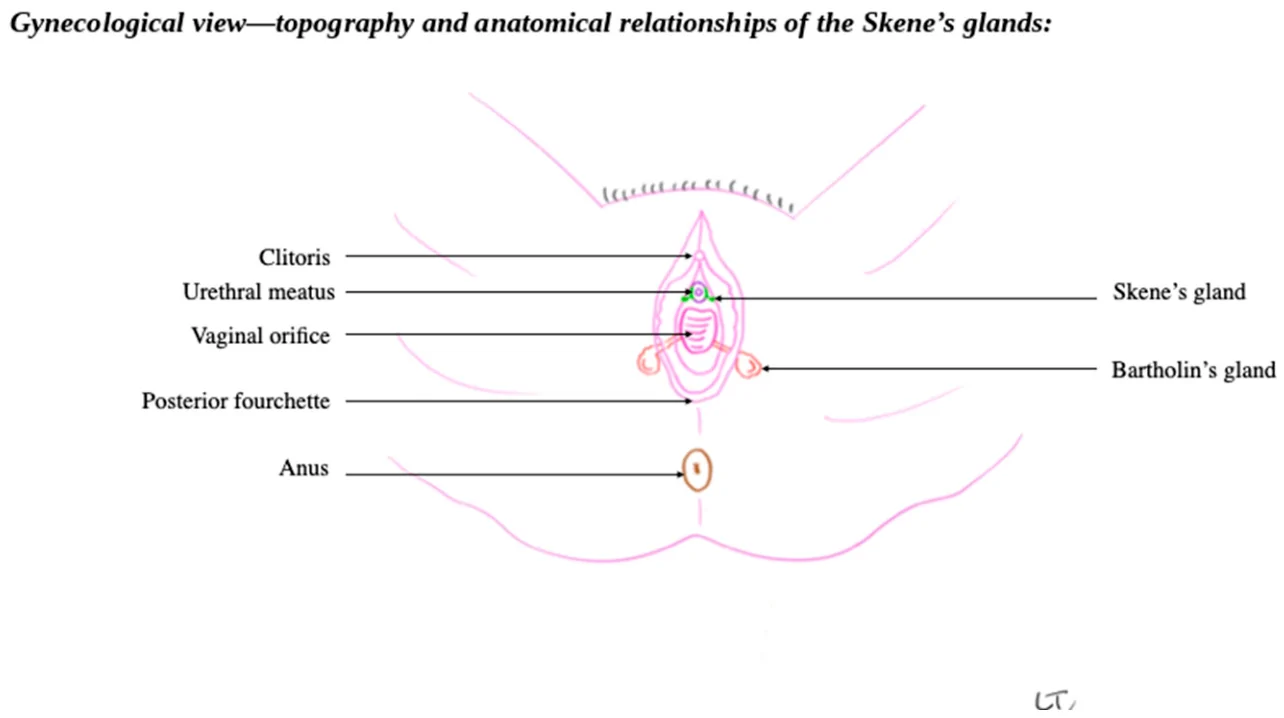
Gynecological (perineal) view showing the topography and anatomical relationships of the Skene’s glands with the clitoris, urethral meatus, vaginal orifice, Bartholin’s glands, posterior fourchette and anus. Original drawing by L.T.; “LT” in the lower right corner is the illustrator’s signature.

**Figure 3:**
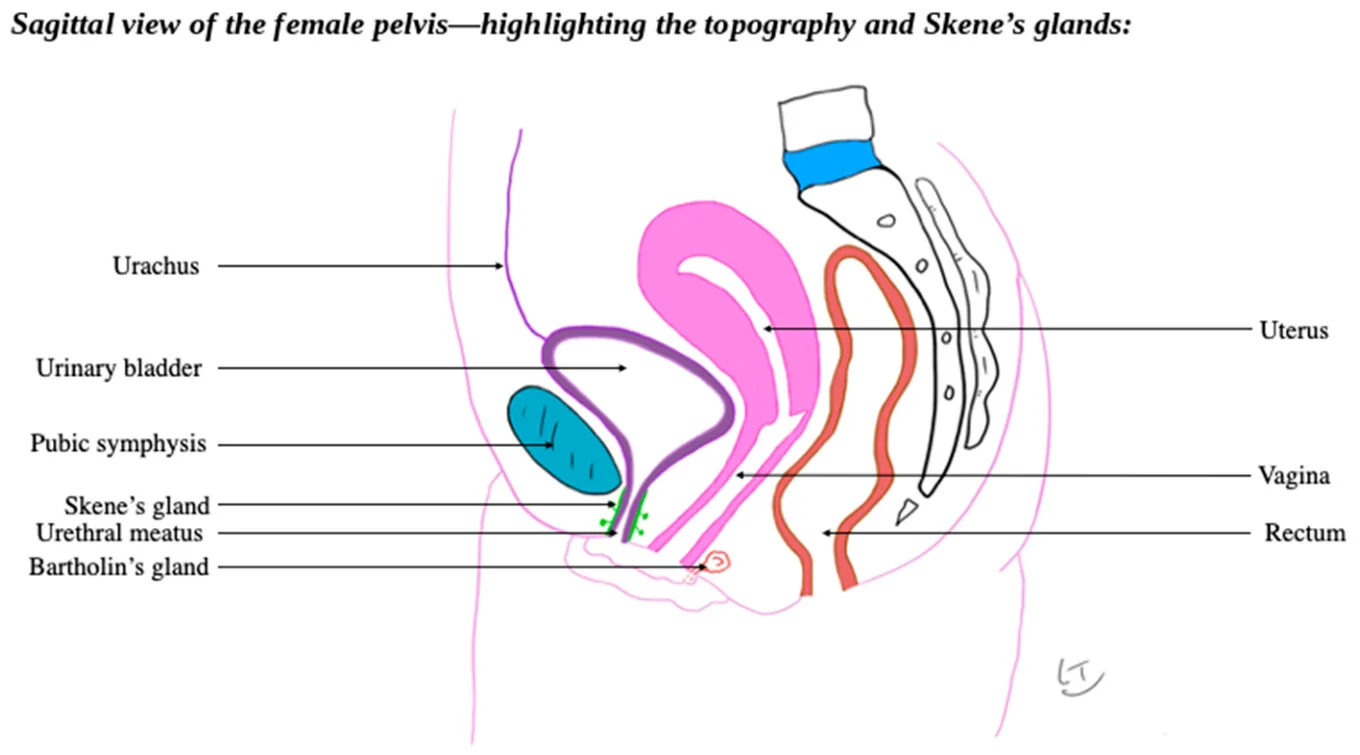
Sagittal view of the female pelvis highlighting the topography of the Skene’s glands in relation to the urethral meatus, urinary bladder, urachus, pubic symphysis, vagina, uterus, Bartholin’s gland and rectum. Original drawing by L.T.; “LT” in the lower right corner is the illustrator’s signature.

Understanding the regional anatomy is critical for interpreting imaging findings and planning radical or organ-preserving surgery. The topographic relationships of the Skene’s glands with the urethral meatus, the vaginal orifice and the neighboring Bartholin’s glands, as well as their position relative to the urinary bladder, urethra, vagina and rectum in the sagittal plane, are illustrated in Figure 2 and Figure 3.

## 4. Clinical Presentation and Paraclinical Assessment

### 4.1. Summary of Published Cases

Because Skene’s gland malignancy is known almost exclusively from isolated case reports and small series, the number of published cases depends on the diagnostic criteria applied. Slopnick et al. identified 15 cases published between 1974 and 2022 (including their own case), restricted to adenocarcinomas resembling prostatic carcinoma, with a median patient age of 71 years (range 46–88); the most frequent presentation was an asymptomatic periurethral or urethral lesion (33.3% of cases), followed by hematuria or vaginal bleeding (20.0%), and serum PSA was elevated in all eight patients in whom it was measured [4]. Applying broader criteria that also accepted non-prostatic phenotypes, Gao et al. counted 20 previously reported cases [2], and Li and Zhang identified 22 cases (median age 70 years, range 46–91; tumor diameter 0.3–9.0 cm, mean 2.6 cm) [5]. Since the latter review, at least three further cases have been published [14,15,16], and two additional reports [7,17] could not be confirmed as included in previous reviews; allowing for overlap between reviews, approximately 25 cases have therefore been reported to date. Table 1 summarizes the individually identifiable cases published since 2015, together with those identified from reference lists, grouped according to the case definition in Section 2.2; earlier cases (1974–2014) are summarized in previous reviews [2,4,5]. As shown in Table 1, reported management has ranged from limited local excision to radical cystectomy, urethrectomy or anterior pelvic exenteration with lymphadenectomy, and outcomes have varied widely, from durable disease-free survival to early death from metastatic disease, reflecting the heterogeneity of tumor stage and phenotype at presentation and the absence of a standardized treatment algorithm.

**Table 1:** Individually reported cases of Skene’s gland malignancy published since 2015 or identified from reference lists, grouped according to the case definition (Section 2.2): (A) cases meeting the case definition; (B) probable cases, attributed to the Skene’s glands by the original authors, with a non-prostatic phenotype or insufficient immunophenotypic data and without demonstrated origin from native glands; (C) female urethral adenocarcinomas discussed in relation to a possible Skene’s gland origin that do not meet the case definition, shown for comparison. Earlier cases (1974–2014) are summarized in [2,4,5]. NR, not reported; NA, not available; NED, no evidence of disease; ADT, androgen deprivation therapy; LND, lymph node dissection; UTI, urinary tract infection; PSA, prostate-specific antigen; PSAP, prostate-specific acid phosphatase; AR, androgen receptor; PIN, prostatic intraepithelial neoplasia; LOH, loss of heterozygosity; FDG, fluorodeoxyglucose. See Section 5.2 and Section 7 for the imaging protocol and differential diagnosis, respectively.

Source	Age (y)	Presentation	Imaging Reported	Histology/Key Immunohistochemistry	Treatment	Outcome/Follow-Up
**A. Cases meeting the case definition**						
Muto et al., 2017 [10]	69	Recurrent tumor at the external urethral meatus	NR	Adenocarcinoma with intestinal differentiation (CDX2+, MUC2+, CK20+, CK7−); PSAP+, AMACR+, PSA−, NKX3.1−; adjacent native paraurethral glands NKX3.1+/PSAP+/AR+	Surgical excision	NR
Tregnago et al., 2018 [8] (*n* = 4)	61–87 (mean 74.5)	Periurethral or urethral polypoid mass; size 1.0–2.0 cm	NR	Prostatic-type acinar adenocarcinoma (3/4; PSA+, P501S+, NKX3.1+, AMACR+); serous-type (1/4; CK7+, ER+, p16+, PAX8−, WT1−); origin from native glands shown with PIN4 cocktail in 3 cases	Limited local excision, negative margins	All alive and NED at mean 40.7 mo (range 4–132)
Maghsoudi et al., 2019 [18]	75	Macroscopic hematuria; urethral and separate bladder lesion; serum PSA 22.82 ng/mL	NR	Prostatic-type adenocarcinoma, PSA+	Surgical resection, clear margins, negative nodes; no adjuvant therapy	PSA undetectable from 6 weeks postoperatively
Kyriazis et al., 2020 [19]	71	Urethral mass	NR	High-grade prostatic-type adenocarcinoma invading periurethral muscle	Urethrectomy	Alive and NED at 31 mo [2]
Kunc & Biernat, 2021 [11]	78	Incidental finding at radical cystectomy for urothelial carcinoma of the bladder	NR	Prostatic-type adenocarcinoma with PIN-like precursor; PSA+, prostein+, AR+	Radical cystectomy (for coexisting urothelial carcinoma)	NR
Kaufman et al., 2021 [6]	69	Vaginal pain, dysuria, nocturia; pelvic lymphadenopathy; serum PSA 60.8 ng/mL	3D multiparametric MRI; fluciclovine F-18 PET (upstaged nodal disease)	Prostatic-type adenocarcinoma (Gleason-pattern 4+5); PSA+, PSAP+, AR+, NKX3.1+	ADT (bicalutamide) followed by surgery with pelvic LND	PSA 60.8 → 9.94 ng/mL within 2 weeks of ADT
Lenz et al., 2021 [12]	73	Recurrent UTI; 3 × 2 cm polypoid lesion at bladder neck/proximal urethra	NR	Cribriform prostatic-type adenocarcinoma; PSA+, PSAP+, NKX3.1+, AMACR+; *PTEN* mutation with LOH	Transurethral resection (positive margins); further treatment declined	NR
Saeed & Osunkoya, 2021 [20]	NA	NA	NA	Skene’s gland adenocarcinoma; reduced PTEN expression, no ERG overexpression	NA	NA
Thum et al., 2017 [21]	NA	Clinically suspected advanced urethral urothelial carcinoma	NR	Adenocarcinoma with prostatic-type morphology and immunophenotype	NR	NR
Slopnick et al., 2022 [4]	76	Enlarging left inguinal mass	NA	Prostatic-type adenocarcinoma (Gleason-pattern 4+4); NKX3.1+; inguinal and obturator nodal metastases	ADT	Well at 24 mo [2]
Wang & Zhou, 2026 [15]	71	Urinary frequency, dysuria; bladder neck tumor at cystoscopy	Cystoscopy	Skene’s gland adenocarcinoma involving the bladder neck; NKX3.1+	Robot-assisted radical cystectomy, ileal conduit, LND; no adjuvant therapy	Good condition at 48 mo (pT3aN1)
**B. Probable cases (non-prostatic phenotype or insufficient immunophenotypic data; origin from native glands not demonstrated)**						
Dentry et al., 2021 [7]	NR	Vaginal/periurethral lesion detected during follow-up of a previous vaginal neuroendocrine tumor	FDG-PET and MRI; MRI-guided biopsy after non-diagnostic surgical biopsy	Skene’s gland adenocarcinoma (immunophenotype NA)	Anterior pelvic exenteration	In remission at 2 years
Bondili et al., 2021 [17]	57	Vaginal mass and spotting	MRI pelvis; CT thorax (lung metastases)	Ductal/papillary and cribriform adenocarcinoma; ER+, AR+, AMACR+, CK7+, CDX2+, vimentin+; PAX8−, WT1−, p40−; serum PSA low; *TBP–RET* fusion	Palliative platinum-based chemotherapy (two lines)	Partial response, then stable disease at about 18 mo
Gao et al., 2022 [2]	54	Vaginal bleeding; anterior vaginal wall and labia majora masses; inguinal lymphadenopathy	Ultrasonography, contrast-enhanced CT and MRI (mass semi-encircling the urethra)	Adenosquamous carcinoma with intestinal differentiation	Neoadjuvant chemotherapy + surgery	Died 46 days postoperatively (recurrence, multiple metastases)
Alla et al., 2024 [14]	45	Recurrent UTI, macroscopic hematuria, dysuria, dyspareunia; bulging anterior vaginal wall	CT urography; pelvic MRI (71 × 45 × 40 mm periurethral mass with urethral epicenter, external iliac lymph nodes); chest and abdominal CT	Mucinous (intestinal-type) adenocarcinoma with clear cell areas; PAX8+, vimentin+, PR+; PSA−, GATA3−, WT1−, p63−, chromogranin−; serum PSA < 0.01 ng/mL; cT3N2M0	Neoadjuvant chemoradiation (46 Gy with weekly cisplatin), followed by reassessment for surgery	NR
Nuha et al., 2026 [16]	62	Periurethral mass	MRI and PET at recurrence	Moderately differentiated adenocarcinoma, gastrointestinal-type (CK20+, CDX2+, focal PSA+; PAX8−, ER/PR−)	Definitive cisplatin chemoradiation; local recurrence treated with brachytherapy, then anterior pelvic exenteration	Alive, NED at 5-year follow-up
**C. Female urethral adenocarcinomas not meeting the case definition (comparators)**						
Satyanarayan et al., 2015 [22]	60	Obstructive voiding, urethral mass	NR	Urethral adenocarcinoma with mucinous features; adjacent intestinal metaplasia	Wide local excision; later radical cystectomy with ileal conduit	No evidence of recurrence
Tian et al., 2023, Case 1 [23]	49	Obstructive voiding	CT abdomen/pelvis	Distal urethral adenocarcinoma	Partial urethrectomy	Disease-free at 2 years
Tian et al., 2023, Case 2 [23]	58	Meatal itching, painless bleeding	NR	Mucinous adenocarcinoma	Local excision only, no adjuvant therapy	Recurrence, inguinal nodal and iliopsoas metastasis over >10 years

### 4.2. Clinical Presentation and Physical Examination

The clinical presentation of Skene’s gland malignancy is nonspecific and overlaps considerably with that of benign periurethral and anterior vaginal wall conditions, which contributes to frequent diagnostic delay [24]. In a large retrospective series of periurethral and anterior vaginal wall masses, the most common presenting complaints were a palpable anterior vaginal wall or periurethral mass, dyspareunia, and lower urinary tract symptoms such as dysuria, urgency, frequency and recurrent urinary tract infection; vaginal discharge, spotting or overt bleeding, and postvoid dribbling were also reported, while malignancy accounted for only a small minority of cases in that cohort [25]. Obstructive symptoms, such as a weak or interrupted urinary stream, straining or incomplete bladder emptying, may occur when a periurethral tumor compresses or infiltrates the urethra [3,22,23]. In the specific context of Skene’s gland malignancy, an asymptomatic periurethral or urethral nodule and hematuria or vaginal bleeding are among the most frequently documented findings [4].

Clinical behavior is heterogeneous. Most prostatic-type tumors in the published series were small (1.0–2.0 cm) and localized at diagnosis [8], and nodal or distant metastases were documented in a minority of cases (4 of 20 in the review by Gao et al.; lymph node metastases in 5 of 22 in the review by Li and Zhang) [2,5] (D). However, tumors up to 9 cm have been described [5], and some patients presented with inguinal or pelvic lymphadenopathy, vulvar or labial deposits, or lung metastases [2,4,6,17] (D). For female urethral adenocarcinoma in general, which is often large and aggressive at presentation and is diagnosed at a metastatic stage in more than half of cases according to some reports [14], spread to the perineal skin, vulva, bladder and vagina and distant metastases to the lung, liver, bone and brain have been reported in advanced stages [2,3] (E).

Initial evaluation should include a focused history addressing the duration and progression of symptoms, prior periurethral surgery or gland drainage, and risk factors for urethral malignancy, followed by a careful pelvic examination. Physical examination should assess the size, consistency, mobility and fixation of any periurethral mass, the presence of ulceration, bleeding or purulent discharge from the urethral meatus (which may be elicited by gentle compression, or “milking”, of the periurethral region), and any extension toward the anterior vaginal wall, bladder neck or vulvar structures. Because the Skene’s glands and their ducts open beside the external urethral meatus, a primary tumor typically presents as a periurethral or anterior vaginal wall nodule adjacent to the meatus; a labial nodule should rather suggest vulvar or Bartholin gland pathology or metastatic spread, as in a reported case in which a labia majora deposit accompanied a periurethral primary [2]. Bartholin gland lesions, located posterolaterally at the vaginal introitus, are usually readily distinguished on inspection and bimanual and speculum examination, although large Bartholin lesions may extend anteriorly and be mislocalized on imaging [26,27]. Examination of the groins can identify inguinal lymphadenopathy in more advanced disease.

Paraclinical work-up typically includes urinalysis and urine culture to exclude infection, together with urine cytology when malignancy is suspected [24]. Because the exact anatomical epicenter of a periurethral mass, and therefore the most appropriate biopsy route, is often established only after imaging, cross-sectional imaging (ultrasonography and, in particular, pelvic MRI) is usually performed before endoscopic biopsy (E, X). Cystourethroscopy then allows direct visualization of the urethral lumen and meatus, assessment for communication with a possible diverticular cavity, and targeted transurethral, transvaginal or image-guided biopsy of any suspicious lesion [24,25]; when conventional biopsy is non-diagnostic, MRI-guided biopsy has been used successfully in Skene’s gland adenocarcinoma [7] (D). In tumors with suspected prostatic-type differentiation, serum prostate-specific antigen may provide additional diagnostic and follow-up value, as discussed further in Section 6 [4,6,18]. Because clinical examination and basic paraclinical testing alone cannot reliably differentiate benign from malignant periurethral disease, imaging, chiefly pelvic MRI, plays a central role in further characterization, as detailed below.

## 5. Multimodal Imaging Characterization

### 5.1. Ultrasonography

Ultrasonography is commonly used as a first-line imaging method in women presenting with periurethral symptoms, mainly because it is accessible, non-invasive, inexpensive, and allows dynamic real-time assessment. In the evaluation of urethral and paraurethral lesions, ultrasound may help establish whether a mass is cystic, solid, or mixed and may provide preliminary information regarding its relationship with the urethra, anterior vaginal wall, and adjacent soft tissues. It can also demonstrate internal debris, septations, wall thickening, calcifications, vascularity or inflammatory changes. Nevertheless, ultrasound has inherent limitations in this anatomical region. The female urethra is short, deeply situated within the pelvic floor and partly obscured by surrounding bony and soft-tissue structures, while bowel gas and patient-related factors may further reduce image quality [26].

Several sonographic approaches may be used, including transvaginal, transperineal, translabial and high-frequency endovaginal ultrasound. These techniques can be particularly useful for the assessment of distal urethral and paraurethral masses, including Skene’s gland cysts, abscesses, urethral diverticula, leiomyomas and other anterior vaginal wall lesions [26,28]. In general, simple cystic lesions appear as well-defined anechoic or hypoechoic structures, whereas complicated cysts or abscesses may show internal echoes, septations, thickened walls, peripheral hyperemia, and adjacent inflammatory changes. Solid lesions, by contrast, may present as hypoechoic or heterogeneous masses, sometimes with detectable vascular signals on color Doppler examination [28]. The sonographic assessment should focus not only on lesion morphology, but also on its exact anatomical epicenter and any possible communication with the urethral lumen [29].

Specific ultrasound descriptions of Skene’s gland malignancy are limited, reflecting the rarity of this tumor; in one reported case, ultrasonography showed a cystic–solid labial deposit and hypoechoic inguinal lymph nodes [2] (D). Based on the available literature on female urethral and periurethral malignancies, suspicious lesions may appear as predominantly solid or mixed cystic-solid masses, with heterogeneous echotexture, irregular margins, and increased or disorganized Doppler vascularity [4,8,28] (E). Additional clinical and imaging features that should raise concern include rapid enlargement, recurrent bleeding, hematuria, ulceration, fixation to adjacent tissues, recurrence after drainage or excision, and signs of extension toward the urethra, anterior vaginal wall or surrounding periurethral soft tissues. However, these findings are not specific for Skene’s gland origin and cannot reliably differentiate Skene’s gland carcinoma from primary urethral carcinoma, carcinoma arising in a urethral diverticulum, vaginal carcinoma or metastatic disease.

Three-dimensional ultrasound may add value by improving spatial visualization of paraurethral and anterior vaginal wall masses. Multiplanar reconstruction can help clarify the relationship between the lesion, the urethra, the vaginal wall and pelvic floor structures, which may be useful for preoperative planning and interdisciplinary communication [30]. Despite this advantage, three-dimensional ultrasound remains primarily an anatomical mapping tool. Its ability to distinguish benign from malignant periurethral lesions is limited, and suspicious findings should be further assessed with cross-sectional imaging and tissue diagnosis.

### 5.2. Magnetic Resonance Imaging

Most vulvar and periurethral disorders are identified and followed clinically, with imaging playing a complementary role. When imaging is required, magnetic resonance imaging (MRI) is the preferred technique because of its superior soft-tissue contrast and high spatial resolution, and it is regarded as the reference imaging modality for urethral and periurethral lesions [13,27,31] (E). Direct MRI evidence in Skene’s gland malignancy is limited to a few case reports. MRI showed a solid mass centered in the anterior vaginal wall that semi-encircled the urethra, resembling the periurethral configuration of the male prostate (a pattern also described by Korytko et al., as cited in [2]), together with a labial deposit and enlarged inguinal lymph nodes [2]; MRI demonstrated a 7 cm sub-vesical periurethral mass with a urethral epicenter, most probably arising from a left periurethral gland, with local extension and external iliac lymph nodes [14]; three-dimensional multiparametric MRI clarified the anatomy of a primary lesion before surgery [6]; and MRI, combined with FDG-PET, detected a Skene’s gland adenocarcinoma during follow-up of a previous vaginal tumor and guided its biopsy [7] (D). The protocol proposed in Table 2 is therefore adapted from recommendations for urethral and vulvar imaging and has not been validated specifically for Skene’s gland tumors. The European Society of Urogenital Radiology recommends imaging protocols incorporating T2-weighted sequences (T2W), diffusion-weighted imaging with Apparent Diffusion Coefficient (ADC) mapping, and dynamic contrast-enhanced imaging [32]. Fat-suppressed T2W sequences are generally preferred because they improve visualization of the primary lesion by enhancing contrast between the tumor and the surrounding perineal adipose tissue. Diffusion-weighted imaging can further aid in delineating the tumor. Imaging protocols dedicated to urethral assessment should incorporate a small field of view and high-resolution T2W sequences acquired in the axial, sagittal, and coronal planes. The use of fat-suppressed techniques may improve visualization of urethral diverticula by increasing lesion conspicuity. T1-weighted (T1W) imaging is particularly valuable for detecting hemorrhagic or protein-rich material within diverticular cavities. In patients with malignancies, additional large field-of-view sequences are useful for evaluating regional lymphadenopathy, bone involvement, and distant metastatic spread [33]. At the same time, MRI also provides valuable postoperative follow-up and surveillance information [31].

### 5.3. Computed Tomography

Computed tomography (CT) has a limited role in the primary local characterization of suspected Skene’s gland malignancy because its soft-tissue contrast is inferior to MRI, particularly in the evaluation of the distal urethra, paraurethral tissues, anterior vaginal wall and pelvic floor. Therefore, CT should not be considered the preferred modality for defining the local epicenter of a small periurethral tumor or for assessing subtle invasion of adjacent soft tissues. Its main value lies in systemic staging, especially in patients with invasive, bulky, recurrent or histologically confirmed malignant disease. Contrast-enhanced CT of the chest, abdomen and pelvis may be used to evaluate pelvic, inguinal and retroperitoneal lymph nodes, detect distant metastases and assess associated abdominal or thoracic disease. CT urography may also be considered when urothelial involvement, synchronous urinary tract lesions or upper-tract disease must be excluded. Thus, in suspected or confirmed Skene’s gland malignancy, CT is best regarded as a complementary staging tool rather than a primary local diagnostic method [36,37]. In reported Skene’s gland cases, contrast-enhanced CT was used for systemic staging and detected lung metastases whose response to chemotherapy was then monitored by CT [2,17] (D).

### 5.4. PET/CT Imaging

Positron emission tomography/computed tomography (PET/CT), most commonly using 18F-fluorodeoxyglucose, provides functional information regarding tumor glucose metabolism and may complement conventional cross-sectional imaging in selected patients. In urethral and periurethral malignancies, PET/CT may be useful for detecting metabolically active lymph node disease, distant metastases or recurrent disease, particularly when CT or MRI findings are equivocal. It may also help distinguish viable tumor from postsurgical or post-treatment anatomical changes, although inflammatory lesions and infection can also show increased tracer uptake and may lead to false-positive findings. In the context of Skene’s gland malignancy, evidence remains very limited and is largely extrapolated from primary urethral carcinoma, female urethral adenocarcinoma and isolated case reports. Therefore, PET/CT should not be considered a routine replacement for pelvic MRI or histopathological confirmation. Its role is more appropriately reserved for selected cases, especially advanced disease, suspected nodal or distant spread, treatment response assessment, or evaluation of recurrence. Further studies are required before PET/CT can be incorporated into standardized staging algorithms for Skene’s gland malignancies [38,39]. In Skene’s gland adenocarcinoma specifically, fluciclovine F-18 PET, a tracer used in prostate cancer, upstaged nodal disease and prompted pelvic lymphadenectomy [6], and FDG-PET combined with MRI identified a lesion that was subsequently confirmed by MRI-guided biopsy [7] (D). Prostate-specific membrane antigen (PSMA)-targeted PET has not, to our knowledge, been reported in this setting but may be considered by analogy in prostatic-type tumors (X).

## 6. Histopathology and Molecular Landscape

Histopathological variants reported in Skene’s gland malignancies include adenocarcinoma resembling prostatic acinar or ductal carcinoma, which is the predominant subtype [4,6,8,12]; adenocarcinoma with intestinal or mucinous differentiation [10,16]; serous-type adenocarcinoma [8]; adenosquamous carcinoma [2]; and ductal/papillary adenocarcinoma with a mixed immunophenotype [17]. Clear cell adenocarcinoma of the female urethra is generally regarded as a distinct, Müllerian-type urethral tumor rather than a Skene’s gland neoplasm [40,41]. Microscopically, Skene’s gland adenocarcinomas demonstrate glandular proliferation with tubular, cribriform, fused or poorly formed glands, sometimes with mucin production, together with cellular atypia and mitotic activity [8].

Skene’s gland adenocarcinoma is a very rare tumor, with only a limited number of documented cases in the literature. Arising from the Skene’s glands, this shares both morphological characteristics and immunohistochemical profiles with prostatic adenocarcinoma. Nevertheless, because of the extremely limited number of reported cases, it remains unclear whether the grading systems currently used for prostate adenocarcinoma can be reliably applied to these tumors [8]. Determining the primary site of origin in female urethral carcinoma is frequently challenging. In the case of Skene’s gland adenocarcinoma with intestinal differentiation, immunohistochemical profiling plays a crucial role in establishing the tumor’s histogenetic origin [10].

Histologically, the Skene’s glands contain secretory epithelium expressing PSA and PSAP, and normal Skene’s gland derivatives are consistently NKX3.1-positive [8,9,15]. In tumors with prostatic-type morphology, PSA, PSAP, NKX3.1, P501S (prostein), AMACR (P504S) and androgen receptor are frequently expressed [6,8,11,12], whereas CK7, CK20, CDX2 and MUC2 may be positive in tumors with intestinal differentiation [2,10,16]. However, no single immunohistochemical marker establishes Skene’s gland origin in isolation, and formal estimates of sensitivity and specificity cannot be derived from the available case reports. PSA positivity is not universal: it has been reported in only about 50–80% of Skene’s gland adenocarcinomas and may be absent in otherwise typical tumors [10,17], and Gao et al. concluded that PSA lacks diagnostic specificity for Skene’s gland carcinoma [2]. The main areas of marker overlap are: (i) other female urethral adenocarcinomas, in which PAX8 identifies a Müllerian-type (including clear cell) subgroup but is also expressed in some tumors attributed to the Skene’s glands [14,40]; (ii) colorectal-type adenocarcinoma, whether primary urethral or metastatic, as CDX2, SATB2, CK20 and MUC2 are shared by intestinal-type Skene’s gland tumors, so that clinical and endoscopic exclusion of a colorectal primary is required [40,42]; (iii) Müllerian tumors, as PAX8, WT1, estrogen and progesterone receptors and vimentin favor a Müllerian lineage, but ER, PR, vimentin, p16 and PAX8 have been reported in serous-type, intestinal-type or mixed tumors attributed to the Skene’s glands [8,14,17]; (iv) urothelial-associated tumors, as GATA3, p63/p40 and a CK7+/CK20+ profile favor urothelial differentiation, whereas p63 with high-molecular-weight cytokeratin, as part of a PIN4 cocktail, also highlights basal cells and can demonstrate origin from pre-existing glands [8,11,41]; (v) neuroendocrine tumors, which may arise in the same region and are identified by chromogranin and synaptophysin [7,41]; and (vi) metastatic adenocarcinoma, including prostatic-type tumors arising elsewhere in the female genital tract [9,42]. AMACR is also expressed in intestinal-type and clear cell adenocarcinomas of the urinary tract and is therefore not specific [17,43]. Immunohistochemistry should thus be interpreted as a pattern (typically including PSA, PSAP, NKX3.1 and AR; CK7, CK20, CDX2 and SATB2; PAX8, WT1 and ER/PR; GATA3 and p63; and neuroendocrine markers when indicated), together with morphology, tumor epicenter on imaging and exclusion of another primary (X). As in prostate carcinoma, serum PSA may be a useful biomarker for monitoring prostatic-type tumors: PSA normalized after definitive treatment in all tested patients in the review by Slopnick et al. [4] became undetectable after resection [18] and fell rapidly under androgen deprivation [6] (D). Rising PSA levels may therefore suggest recurrence before clinical symptoms develop [11,15].

Molecular data are limited to isolated observations and do not yet allow a molecular characterization of the entity. Next-generation sequencing of one prostatic-type case identified a mutation of the phosphatase and tensin homolog (*PTEN*) gene with loss of heterozygosity (LOH) [12]; another case showed reduced PTEN expression without ERG overexpression [20]; and a *TBP–RET* fusion was reported in a metastatic case with a mixed immunophenotype, although orthogonal confirmation was not possible [17]. *PTEN* mutations have also been reported in about 15% of female urethral adenocarcinomas overall [41]. Overexpression of *AMACR* (P504S) is characteristic of prostatic adenocarcinoma, whereas benign prostatic tissue shows absent or minimal immunoreactivity [43], but it is not specific for Skene’s gland origin [17]. These single-case findings should be regarded as hypothesis-generating.

## 7. Differential Diagnosis of Skene’s Gland Malignancies

Because of the intimate anatomical relationship between the distal urethra, paraurethral glands, anterior vaginal wall and vulvar soft tissues, the differential diagnosis of Skene’s gland malignancy can be difficult [44,45]. Benign cystic or inflammatory lesions may present with symptoms similar to those of malignant tumors, including a palpable periurethral mass, dysuria, dyspareunia, recurrent infection, hematuria, or obstructive urinary complaints. MRI plays an important role in the initial characterization of these lesions by identifying the precise anatomical origin, the relationship with the urethral lumen, the presence of a communicating tract, and the internal structure of the mass [44,45,46]. Features such as solid enhancing components, diffusion restriction, irregular margins, or invasion of adjacent tissues may suggest malignancy, but they are not specific for Skene’s gland origin. Primary urethral carcinoma, carcinoma arising within a urethral diverticulum, anterior vaginal wall carcinoma and metastatic adenocarcinoma may show overlapping imaging appearances. Therefore, MRI findings should be integrated with clinical examination, cystourethroscopy, histopathological assessment and immunohistochemical profiling [4,44,45,47]. The entities that should be considered in the differential diagnosis are summarized in Table 3, grouped into benign (including inflammatory and iatrogenic) lesions, primary malignant tumors and secondary malignant involvement, and should be interpreted in conjunction with the topographic relationships illustrated in Figure 2 and Figure 3.

## 8. Therapeutic Management

### 8.1. Surgical Treatment

In this pathology, surgery remains the cornerstone of treatment. The choice of surgical management is influenced by multiple tumor-related factors such as tumor dimensions, depth and extent of local invasion, degree of urethral and periurethral involvement, presence of adjacent organ infiltration, and overall disease stage. In addition, patient-related factors such as age, performance status, comorbid conditions, urinary function, and anticipated postoperative quality of life must be carefully considered when planning treatment. Because malignant tumors of the Skene’s glands are exceptionally uncommon, there is no universally accepted surgical protocol. In the reported Skene’s gland cases, small (≤2 cm), localized prostatic-type tumors were most often treated by local excision with negative margins, without recurrence during follow-up; of the 15 cases reviewed by Slopnick et al., eight were treated by local excision, two by radical surgical resection, two with radiotherapy and one with adjunctive androgen deprivation [4,8] (D). The more extensive procedures described below are largely extrapolated from female urethral carcinoma (E) or reflect expert inference based on tumor extent (X); the evidence basis of each recommendation is summarized in Table 4. Complete resection with negative margins is the primary objective, and management is therefore individualized according to tumor extent, ranging from organ-preserving local excision for limited lesions to radical urethrectomy or anterior pelvic exenteration in advanced invasive disease [10,37].

Urethra-sparing endoscopic approaches, such as transurethral resection and laser ablation or vaporization of small, superficial tumors of the distal urethra and meatus, are described in the management of primary urethral carcinoma, mainly in men [36,37] (E). Their role in Skene’s gland malignancy is uncertain: because these tumors arise in the periurethral glands beneath the urothelium rather than from the mucosal surface, endoscopic resection may not achieve clear margins, as illustrated by a transurethrally resected Skene’s gland adenocarcinoma with positive margins [12] (D). Endoscopic approaches may therefore be regarded mainly as diagnostic or palliative options, or as definitive treatment only for small, superficial lesions in patients unfit for open excision (X).

Radical urethrectomy is typically reserved for advanced or recurrent lesions showing infiltration of the proximal urethra or spread to adjacent periurethral and vaginal tissues. Surgical management can involve total urethral excision combined with removal of involved surrounding soft tissues and, in selected cases, partial excision of the anterior vaginal wall. Such an aggressive operative strategy may enhance local tumor containment in high-risk disease [3] (E). Lymphadenectomy may be indicated in cases with radiologically suspicious nodal involvement, advanced tumor stage, or high-grade adenocarcinoma histology. The pattern of nodal spread varies according to tumor location, with distal lesions more commonly associated with inguinal lymphatic dissemination, whereas proximal tumors tend to involve the pelvic nodal chains [2] (E). Anterior pelvic exenteration or radical cystectomy is generally reserved for extensive disease characterized by deep local invasion, involvement of the bladder neck, widespread extension into periurethral tissues, or regional lymph node metastases, and has been used in Skene’s gland cases involving the bladder neck or after failure of radiotherapy [7,15,16] (D).

No specific staging system exists exclusively for Skene’s gland malignancies, and FIGO staging is not applicable; most authors apply the TNM/AJCC staging used for female urethral carcinoma, whose limitations for tumors of the anterior urethral wall have led to the proposal of histology-based staging systems [41] (E). Multiple factors, including tumor anatomical location and size, histopathological subtype, extent of tissue infiltration, lymph node involvement, surgical margin status, and presence of distant metastases, influence the clinical outcome of urethral carcinoma. Lesions arising in the anterior (distal) urethra generally demonstrate more favorable outcomes than tumors involving the posterior (proximal) urethra [10,37]. Early-stage localized disease appears associated with favorable outcomes after complete surgical excision, whereas advanced tumors with nodal dissemination demonstrate a poorer prognosis [10,37] (Section 8.4).

### 8.2. Adjuvant Therapy

Radiotherapy may be considered in the management of Skene’s gland carcinoma as an adjuvant treatment for locally advanced disease, as well as a definitive therapeutic option in patients who are unsuitable for radical surgery or in whom extensive surgical procedures would result in substantial morbidity [10,37] (E). In selected cases, radiation-based approaches, with or without concurrent chemotherapy, may contribute to local tumor control and symptomatic relief. In a locally advanced, node-positive tumor (cT3N2M0), neoadjuvant chemoradiation with weekly cisplatin was chosen before planned surgery [14]; in another case, definitive chemoradiation with cisplatin achieved a complete clinical response but was followed by local recurrence treated with interstitial brachytherapy; subsequent radiation-induced necrosis and osteomyelitis eventually required anterior pelvic exenteration [16] (D). Because malignant tumors originating from the Skene’s glands are exceptionally rare, current evidence is derived primarily from isolated case reports and extrapolation from female urethral carcinoma series, and standardized radiotherapy protocols have not yet been established [2,15,23].

Data concerning the efficacy of systemic chemotherapy in Skene’s gland carcinoma remain uncertain because of the exceptionally low incidence of this neoplasm. Consequently, chemotherapeutic strategies have largely been adapted from protocols established for urothelial malignancies and other adenocarcinomas of the female genital and urinary tracts. In advanced, metastatic, or recurrent settings, platinum-containing combinations have most frequently been employed [15,23,47] (E); in a metastatic Skene’s gland adenocarcinoma, docetaxel–carboplatin followed by gemcitabine–carboplatin produced a partial response and then disease stabilization [17] (D). In prostatic-type tumors, androgen deprivation therapy has produced marked PSA responses and has been used as the main or neoadjuvant treatment in node-positive disease [4,6] (D).

### 8.3. Follow-Up and Surveillance

At present, no standardized surveillance recommendations have been established for patients with Skene’s gland adenocarcinoma owing to the rarity of the disease and the limited number of reported cases. Consequently, follow-up should rely on comprehensive clinicopathological assessment integrating clinical findings, imaging studies, and biomarker evaluation. In tumors demonstrating prostatic differentiation, serial prostate-specific antigen (PSA) measurements may provide additional value when interpreted alongside radiologic and physical examination findings [11]. Available evidence suggests that disease relapse most commonly develops within the pelvic region; however, the actual incidence of recurrence remains difficult to determine because long-term outcome data are sparse. Current follow-up strategies are generally individualized and may include scheduled pelvic examinations, pelvic magnetic resonance imaging (MRI), cystourethroscopic evaluation when clinically indicated, and computed tomography (CT) imaging in patients with advanced or high-risk disease [2,15,23,37].

### 8.4. Prognosis and Outcomes

Evidence on prognosis is limited to case-level follow-up, which is often short or unreported, so that disease-specific survival for Skene’s gland malignancy cannot be estimated. In the systematic review by Slopnick et al., which included only prostatic-type adenocarcinomas, no recurrence or cancer-related death occurred during a median follow-up of 11.5 months, and PSA normalized after definitive treatment in all tested patients [4]. In the four-case series by Tregnago et al., tumors measured 1.0–2.0 cm were excised with negative margins and remained free of recurrence after a mean follow-up of 40.7 months (range 4–132) [8]. In the broader review by Li and Zhang, follow-up was available for 11 of 22 patients, of whom two died and one recurred, and lymph node metastases were present in five patients [5] (D).

Taken together with the individual reports in Table 1, the following factors appear relevant, although none can be quantified: (i) local recurrence, observed after local excision alone and after definitive chemoradiation [16], and a concern after transurethral resection with positive margins [12]; (ii) nodal metastasis, inguinal in distal and pelvic in proximal tumors [2], present at diagnosis in several of the more aggressive cases [2,4,6]; (iii) distant metastasis to the lung, liver, bone or abdominal viscera, associated with death or palliative treatment [2,17]; (iv) tumor size, with uniformly favorable outcomes in small (≤2 cm) localized tumors [8], whereas tumors up to 9 cm have been reported [5]; (v) margin status, negative margins having been achieved in the cases with durable disease-free survival [8,18]; (vi) urethral and bladder-neck involvement, which required urethrectomy, cystectomy or exenteration [7,15,16,19]; and (vii) histological subtype, the most aggressive courses having been observed in tumors with non-prostatic (adenosquamous, intestinal-type or mixed) phenotypes [2,16,17], whereas prostatic-type tumors were mostly localized, although a prostatic-type case with widespread metastases has also been described [2] (D). By extrapolation from primary urethral carcinoma, advanced stage, nodal involvement, non-urothelial histology and proximal location are associated with worse survival; five-year relative overall survival for urethral carcinoma in Europe is approximately 54%, and women tend to present with more advanced disease [3,37] (E).

**Table 4:** Main diagnostic and management statements and their evidence basis. D, direct evidence from reported Skene’s gland malignancies; E, evidence extrapolated from female urethral carcinoma, female urethral adenocarcinoma or benign periurethral lesions; X, expert inference based on anatomy and tumor extent.

Statement	Evidence Basis	Key References
Consider Skene’s gland malignancy in any solid or complex periurethral mass, particularly in postmenopausal women with hematuria, vaginal bleeding or obstructive symptoms	D	[2,4,5]
Ultrasonography as the first imaging test to detect a paraurethral mass and distinguish cystic from solid components	E	[26,28,30]
Pelvic MRI as the preferred modality for tumor epicenter, urethral, periurethral and anterior vaginal wall involvement and regional nodes	E (supported by D in few cases)	[2,6,7,13,14,27,31]
Imaging before endoscopic biopsy; cystourethroscopy with targeted biopsy for tissue diagnosis; MRI-guided biopsy when conventional biopsy is non-diagnostic	E, X; D for MRI-guided biopsy	[7,24,25]
Contrast-enhanced CT of chest, abdomen and pelvis for systemic staging	E (D in few cases)	[2,17,36,37]
PET/CT (FDG or prostate-cancer tracers) in selected advanced, recurrent or equivocal cases	E, D, X	[6,7,38,39]
Immunohistochemistry interpreted as a pattern; no single marker proves Skene’s gland origin	D, E	[8,10,17,40,41,42]
Serum PSA for diagnosis support and monitoring of prostatic-type tumors	D	[4,6,18]
Local excision with negative margins for small, localized tumors	D	[4,8,18]
Radical urethrectomy, cystectomy or anterior pelvic exenteration for extensive or bladder-neck disease	E, D	[3,7,15,16,36,37]
Lymphadenectomy for radiologically suspicious nodes, advanced stage or high-grade histology	E, D	[2,6,15,37]
Radiotherapy or chemoradiation for unresectable or locally advanced disease; platinum-based chemotherapy for metastatic disease	E, D	[14,16,17,37,47]
Androgen deprivation therapy in advanced prostatic-type tumors	D, X	[4,6]
Follow-up with examination, pelvic MRI, cystourethroscopy when indicated, CT in high-risk disease and PSA in prostatic-type tumors	E, X	[2,11,15,23,37]

## 9. Current Challenges and Future Perspectives

Previous syntheses of this entity have generally taken the form of a single new case report accompanied by a literature review, most notably the systematic review and case report by Slopnick et al. (15 prostatic-type cases, 1974–2022) [4], the case report and review by Gao et al. (20 cases) [2], the systematic review by Li and Zhang (22 cases) [5] and the more recent case report and review by Wang and Zhou (2026) [15]. The present review differs in scope and organization rather than in the underlying evidence base, which remains the same small pool of published cases. Rather than being structured around a single index case, it is organized as a modality-by-modality diagnostic framework, moving systematically through clinical and paraclinical assessment, ultrasonography, MRI (including a proposed imaging protocol), CT and PET/CT, before addressing histopathology and an anatomically anchored differential diagnosis of thirteen mimicking entities grouped as benign, primary malignant and secondary malignant (Table 3). It also applies an explicit case definition and a reproducible multi-database search to update the case summary through 2026 (Table 1), and makes explicit whether each recommendation derives from Skene’s gland cases or from female urethral carcinoma (Table 4). This imaging- and differential-diagnosis-centered structure, rather than new clinical data, is intended to be the review’s principal contribution.

Several challenges continue to limit the current understanding and clinical management of Skene’s gland malignancies. First, because these tumors are exceptionally uncommon, robust evidence-based guidelines are lacking. Current management strategies are largely based on individual case reports or adapted from treatment approaches used for female urethral carcinoma.

Inconsistent terminology in the literature complicates accurate interpretation and comparison of published data. While some investigators describe these lesions as paraurethral adenocarcinomas, others categorize them as subtypes of urethral adenocarcinoma.

Available molecular data remain scarce. Further genomic and proteomic research may improve tumor classification and facilitate the identification of potential therapeutic targets. In addition, emerging technologies such as artificial intelligence-based imaging analysis and radiomic assessment could enhance the distinction between benign and malignant periurethral lesions.

The establishment of international registries and multicenter collaborative databases could significantly enhance epidemiological knowledge and support the development of standardized therapeutic guidelines.

## 10. Limitations

This review has several limitations. First, although the literature search was structured, documented and reproducible across three databases, this is a narrative rather than a systematic review: no protocol was registered, no formal risk-of-bias assessment was performed, database searches were limited to 2015–2026 with earlier reports identified through reference lists and previous reviews, and some eligible reports, particularly those in non-English languages or older, non-indexed literature, may have been missed. Second, the evidence base itself is inherently limited: as summarized in Table 1, almost all available data derive from single case reports or very small case series, with substantial heterogeneity in reported histology, immunohistochemical panels, imaging, treatment and length of follow-up, and with outcome data missing or incompletely reported in several cases. This precluded any pooled or quantitative synthesis, such as a meta-analysis of treatment outcomes or survival. Third, the case definition applied here is pragmatic and has not been validated; because Skene’s gland malignancy is frequently indistinguishable from other female urethral adenocarcinomas without detailed immunohistochemical and molecular work-up, some cases classified as Skene’s gland origin in the literature, particularly those with non-prostatic phenotypes, may in fact represent primary urethral or paraurethral tumors of a different histogenesis, which could affect the applicability of the imaging and management data summarized here. Fourth, most imaging and management recommendations are extrapolated from female urethral carcinoma (Table 4). Finally, the imaging descriptions in this review are derived from the literature rather than from a dedicated, systematic pictorial analysis of primary MRI, ultrasound or histopathology images; consequently, the anatomical figures accompanying this review (Figure 2 and Figure 3) are original schematic illustrations rather than diagnostic imaging examples. These limitations underscore the need for prospective, multicenter data collection, as outlined in the Current Challenges and Future Perspectives Section above.

## 11. Conclusions

Skene’s gland malignancies are exceptionally rare tumors of the female periurethral region, and their diagnosis remains challenging because clinical presentation is often nonspecific and overlaps with benign paraurethral, urethral, and anterior vaginal wall disorders. A directed clinical history and pelvic examination are the first steps; imaging should then define the lesion before endoscopic or image-guided biopsy, and its findings determine staging and the choice of treatment.

Ultrasonography may be useful as an initial and complementary examination, particularly for detecting paraurethral lesions, distinguishing cystic from solid components, assessing vascularity with Doppler techniques and guiding selected interventional procedures. However, sonographic findings are not sufficiently specific to confirm malignancy or define the full extent of disease.

MRI remains the key imaging modality for local evaluation, as it provides superior soft-tissue contrast and allows precise assessment of tumor epicenter, relationship with the urethral lumen, periurethral extension, involvement of the anterior vaginal wall and invasion of adjacent pelvic structures. CT and PET/CT may offer additional value in selected cases, mainly for nodal assessment, detection of distant disease, evaluation of recurrence and staging of advanced tumors. Nevertheless, imaging findings must always be interpreted in conjunction with clinical examination, cystourethroscopy and pathological assessment.

Definitive diagnosis requires histopathological confirmation. Immunohistochemistry (PSA, PSAP and NKX3.1, together with a broader panel to exclude urethral, Müllerian, intestinal-type and metastatic adenocarcinomas) supports, but cannot by itself prove, a paraurethral gland origin. Local excision with negative margins appears adequate for small localized prostatic-type tumors, whereas radical surgery, radiotherapy, chemotherapy, androgen deprivation or combined strategies are reserved, largely by extrapolation from female urethral carcinoma, for locally advanced, node-positive, recurrent or aggressive disease.

Given the rarity of these tumors and the absence of standardized management guidelines, individualized treatment planning within a multidisciplinary team is essential. Larger multicenter studies and pooled analyses are needed to refine imaging-based diagnostic algorithms, identify prognostic factors, and develop evidence-based recommendations for staging, treatment, and follow-up.

## Figures and Tables

**Table 2:** Proposed magnetic resonance imaging protocol for the evaluation of female urethral and periurethral lesions [31,34,35]. The protocol is extrapolated from female urethral and vulvar imaging recommendations (evidence level E) and has not been validated specifically in Skene’s gland malignancy. Abbreviations: MRI, magnetic resonance imaging; FOV, field of view; T1W, T1-weighted; T2W, T2-weighted.

Imaging Component	Recommended Protocol	Diagnostic Value
**MRI system**	**Examination performed on a 1.5-T or 3-T scanner using a pelvic phased-array or multichannel cardiac coil**	**Provides high spatial resolution and optimal soft-tissue contrast for assessment of urethral and periurethral anatomy.**
**Essential sequences**	**High-resolution, thin-section T2W images acquired in axial, sagittal, and coronal planes with a small field of view centered on the urethra**	**Defines urethral anatomy, lesion location, tumor size, and local extension into adjacent tissues.**
**Pelvic overview**	**Large field-of-view (FOV) sagittal T2W imaging of the pelvis**	**Evaluates involvement of neighboring pelvic organs, pelvic floor structures, and surrounding soft tissues.**
**Extended assessment**	**Large FOV pelvic and lower abdominal imaging when regional extension or nodal disease is suspected**	**Facilitates detection of regional disease extension, pelvic lymphadenopathy, and associated abnormalities.**
**Complementary anatomical imaging**	**High-resolution fat-suppressed axial T2W images together with axial T1W pelvic sequences**	**Improves characterization of periurethral tissues and identification of hemorrhage or proteinaceous components.**
**Contrast-enhanced imaging**	**Fat-suppressed T1W images obtained before and after intravenous gadolinium administration; subtraction images when appropriate**	**Improves delineation of tumor margins, evaluates enhancement characteristics, and assists in detecting invasion of adjacent organs.**

**Table 3:** Differential diagnosis of Skene’s gland malignancies, grouped into benign lesions, primary malignant tumors and secondary malignant involvement: anatomical and MRI features. Abbreviations: MRI, magnetic resonance imaging; T1W, T1-weighted; T2W, T2-weighted; IHC, immunohistochemistry; PSA, prostate-specific antigen; PSAP, prostate-specific acid phosphatase; NKX3.1, NK3 homeobox 1; CDX2, caudal-type homeobox 2; SATB2, special AT-rich sequence-binding protein 2; PAX8, paired box gene 8.

Pathology	Nature	Anatomic Location/Relationship	MRI Features	Key Distinguishing Features
**Benign lesions (including inflammatory and iatrogenic mimics)**				
Urethral diverticulum	Benign	Periurethral outpouching communicating with the urethral lumen, usually posterolateral or circumferential around the mid urethra	Cystic lesion, usually T2-hyperintense and T1-hypointense; may be round, oval, U-shaped, horseshoe, or “saddlebag”	Identification of a neck/ostium communicating with the urethra is the key feature. Complex content, stones, mural irregularity or an enhancing mural nodule should raise suspicion for complication or malignant transformation. [26,27,34,48]
Skene’s gland cyst/abscess	Benign/inflammatory	Distal paraurethral lesion, typically below the pubic symphysis and near the external urethral meatus; usually no communication with urethral lumen	Simple cyst: T1-hypointense, T2-hyperintense, thin wall. Abscess or proteinaceous/hemorrhagic content: variable signal, including T1 hyperintensity and T2 hypointensity; rim enhancement may occur	Distal location, teardrop/oval morphology, and absence of urethral communication help differentiate it from urethral diverticulum. Infection may mimic malignancy clinically and radiologically. [26,27,46]
Gartner duct cyst	Benign	Usually anterolateral upper or mid vaginal wall; separate from the urethra	Usually T1-hypointense and T2-hyperintense; signal may vary with proteinaceous or hemorrhagic content	Location in the vaginal wall rather than true periurethral tissue is the main discriminator. May be associated with urinary tract anomalies. [26,27]
Müllerian duct cyst/epidermal inclusion cyst	Benign	Vaginal wall cysts; Müllerian cysts usually upper/mid vagina; epidermal inclusion cysts often lower posterior/lateral vaginal wall, frequently post-traumatic or postsurgical	Usually well-defined cystic lesion; T2-hyperintense; T1 signal variable depending on content	Large vaginal cysts can be mislocalized as periurethral lesions. [26,27]
Bartholin gland cyst/abscess	Benign/inflammatory; rarely malignant	Posterolateral vaginal introitus, inferior to the perineal membrane; vulvar rather than periurethral	Simple cyst: T1 low/intermediate, T2 high. Abscess: thick enhancing wall, inflammatory changes. Solid component should raise suspicion for malignancy	Usually readily distinguished clinically by its posterolateral location; included because large lesions may extend anteriorly and be mislocalized on imaging. [26,27]
Urethral caruncle/urethral prolapse	Benign	Distal urethral meatus, classically posterior lip of the external meatus; more frequent in postmenopausal women	Small polypoid lesion; may be T1-hypointense, T2-hyperintense, enhance after contrast; usually no aggressive invasion	Clinical inspection is often diagnostic; mucosal prolapse may clinically mimic urethral malignancy. [27,45]
Urethral or periurethral leiomyoma	Benign solid tumor	Urethral, periurethral, or anterior vaginal wall; may protrude toward the vaginal lumen	Well-circumscribed solid mass; T1 low; T2 variable, often low to intermediate if fibromuscular; usually homogeneous enhancement; typically no restricted diffusion	Smooth borders, noninfiltrative behavior, and absence of aggressive features help differentiate it from carcinoma. [27,45]
Periurethral bulking agent/postoperative change	Benign/iatrogenic mimic	Prior anti-incontinence procedure; periurethral or bladder-neck region	Variable signal depending on injected material; may appear nodular or mass-like	Prior procedure is the key discriminator. [26,27]
**Primary malignant tumors**				
Skene’s gland malignancy/Skene’s gland adenocarcinoma	Malignant (primary)	Usually periurethral, close to the distal urethra/external urethral meatus, or at the bladder neck; Skene’s glands are homologous to the male prostate	Solid or complex periurethral mass; usually low to intermediate signal on T1W, intermediate signal on T2W; may semi-encircle the urethra in a prostate-like configuration; diffusion restriction and contrast enhancement may be present [2]	Diagnosis requires histology and immunohistochemistry interpreted as a pattern; no single marker is diagnostic (Section 6). Prostatic-type differentiation may show PSA, PSAP, NKX3.1 and/or androgen receptor positivity; intestinal, serous and adenosquamous patterns have also been reported. [2,4,8,23,40]
Primary urethral carcinoma	Malignant (primary)	Arises from urethral mucosa/wall; may involve distal, mid, or proximal urethra	Solid lesion, usually T1-low and T2-intermediate; diffusion restriction and heterogeneous enhancement are common; may disrupt normal urethral zonal anatomy	Anatomical proximity makes distinction from Skene’s gland malignancy difficult. MRI helps define local extent, but histology/IHC is required for final classification. [27,41,45]
Carcinoma arising in a urethral diverticulum	Malignant (primary)	Tumor developing within or adjacent to a pre-existing urethral diverticulum	Enhancing solid mural nodule or irregular wall thickening within a diverticulum; diffusion restriction; possible periurethral invasion	Important malignant mimic of Skene’s gland malignancy. It is often adenocarcinoma, but urothelial and squamous histologies are also described. [27,34,44,45]
Anterior vaginal wall carcinoma/vaginal carcinoma with urethral involvement	Malignant (primary, vaginal)	Centered in the anterior vaginal wall, with possible secondary urethral invasion	T1-low, T2-intermediate solid lesion; enhancement and restricted diffusion; may invade urethra or periurethral tissues	Epicenter in the vaginal wall rather than periurethral gland/urethral lumen is the key MRI clue. [27,45]
**Secondary malignant involvement**				
Secondary urethral involvement/metastatic adenocarcinoma	Malignant (secondary)	Direct extension or metastasis from colorectal, gynecologic, bladder, or other pelvic malignancy	MRI may overlap with primary urethral or Skene-region carcinoma: solid enhancing lesion, intermediate T2 signal, diffusion restriction	Radiology alone is usually insufficient. Clinical history, endoscopy, systemic staging and IHC are required. CDX2/SATB2 suggest intestinal differentiation; PAX8 may support Müllerian/renal/thyroid lineage but can also appear in some female urethral adenocarcinomas. [27,40,42]

## Data Availability

The original contributions presented in this study are included in the article. Further inquiries, including requests for the search strategies, search dates, record counts and screening decisions, can be directed to the corresponding author.

## Abbreviations

The following abbreviations are used in this manuscript:

MRI	Magnetic Resonance Imaging
US	Ultrasonography
CT	Computed Tomography
PET	Positron Emission Tomography
T1W/T2W	T1-weighted/T2-weighted (MRI sequence)
DWI	Diffusion-Weighted Imaging
ADC	Apparent Diffusion Coefficient
FOV	Field of View
PSA	Prostate-Specific Antigen
PSAP	Prostate-Specific Acid Phosphatase
IHC	Immunohistochemistry
CK7/CK20	Cytokeratin 7/Cytokeratin 20
NKX3.1	NK3 Homeobox 1
AR	Androgen Receptor
CDX2	Caudal-Type Homeobox 2
SATB2	Special AT-Rich Sequence-Binding Protein 2
PAX8	Paired Box Gene 8
*PTEN*	Phosphatase and Tensin Homolog
LOH	Loss of Heterozygosity
AMACR	α-Methylacyl-CoA Racemase (P504S)
ADT	Androgen Deprivation Therapy
AJCC	American Joint Committee on Cancer
ER/PR	Estrogen Receptor/Progesterone Receptor
FDG	18F-Fluorodeoxyglucose
GATA3	GATA Binding Protein 3
LND	Lymph Node Dissection
MUC2	Mucin 2
NA	Not Available
NED	No Evidence of Disease
NR	Not Reported
PIN	Prostatic Intraepithelial Neoplasia
PRISMA	Preferred Reporting Items for Systematic Reviews and Meta-Analyses
PSMA	Prostate-Specific Membrane Antigen
TNM	Tumor, Node, Metastasis
UTI	Urinary Tract Infection
WT1	Wilms Tumor 1
